# Mapping of genetic loci that modulate differential colonization by *Escherichia coli* O157:H7 TUV86-2 in advanced recombinant inbred BXD mice

**DOI:** 10.1186/s12864-015-2127-7

**Published:** 2015-11-16

**Authors:** Lisa M. Russo, Nourtan F. Abdeltawab, Alison D. O’Brien, Malak Kotb, Angela R. Melton-Celsa

**Affiliations:** Department of Microbiology and Immunology, Uniformed Services University of the Health Sciences, Bethesda, MD USA; University of Cincinnati College of Medicine & Cincinnati VA Medical Center, Cincinnati, OH USA; Department Microbiology and Immunology, Faculty of Pharmacy, Cairo University, Cairo, Egypt; Department of Basic Biomedical Sciences, University of North Dakota, Grand Forks, ND USA

**Keywords:** QTL mapping, *E. coli*, O157:H7, Hemorrhagic colitis, BXD strains, Systems genetics, Genetics of susceptibility to infection, Host-pathogen interaction, Genomic biomarkers, Bioinformatics

## Abstract

**Background:**

Shiga toxin (Stx)-producing *E. coli* (STEC) are responsible for foodborne outbreaks that can result in severe human disease. During an outbreak, differential disease outcomes are observed after infection with the same STEC strain. One question of particular interest is why some infected people resolve infection after hemorrhagic colitis whereas others progress to the hemolytic uremic syndrome (HUS). Host age and infection dose have been implicated; however, these parameters do not appear to fully account for all of the observed variation in disease severity. Therefore, we hypothesized that additional host genetic factors may play a role in progression to HUS.

**Methods and Results:**

To mimic the genetic diversity in the human response to infection by STEC, we measured the capacity of an O157:H7 outbreak isolate to colonize mouse strains from the advanced recombinant inbred (ARI) BXD panel. We first infected the BXD parental strains C57BL/6 J (B6) and DBA/2 J (D2) with either 86–24 (Stx2a+) or TUV86-2, an Stx2a-negative isogenic mutant. Colonization levels were determined in an intact commensal flora (ICF) infection model. We found a significant difference in colonization levels between the parental B6 and D2 strains after infection with TUV86-2 but not with 86–24. This observation suggested that a host factor that may be masked by Stx2a affects O157:H7 colonization in some genetic backgrounds. We then determined the TUV86-2 colonization levels of 24 BXD strains in the ICF model. We identified several quantitative trait loci (QTL) associated with variation in colonization by correlation analyses. We found a highly significant QTL on proximal chromosome 9 (12.5–26.7 Mb) that strongly predicts variation in colonization levels and accounts for 15–20 % of variance. Linkage, polymorphism and co-citation analyses of the mapped region revealed 36 candidate genes within the QTL, and we identified five genes that are most likely responsible for the differential colonization.

**Conclusions:**

The identification of the QTL on chromosome 9 supports our hypothesis that individual genetic makeup affects the level of colonization after infection with STEC O157:H7.

**Electronic supplementary material:**

The online version of this article (doi:10.1186/s12864-015-2127-7) contains supplementary material, which is available to authorized users.

## Background

Shiga toxin (Stx)-producing *E. coli* (STEC) are Gram-negative enteric pathogens associated with foodborne outbreaks. *E. coli* O157:H7 accounts for the greatest incidence of disease due to a single STEC serotype in the in the United States. Indeed, *E. coli* O157:H7 is estimated to be responsible for 63,000 of the estimated 175,000 STEC cases per year [1]. Stx, the primary STEC virulence factor, is an AB5 toxin that inhibits protein synthesis and leads to cell death [2]. The A subunit is responsible for the catalytic activity of the molecule while the B pentamer binds to the host cell receptor, globotriaosylceramide (Gb3). An STEC strain may encode Stx1a and/or Stx2a, two biologically similar though antigenically distinct toxins. Some STEC strains also have the locus of enterocyte effacement (LEE) pathogenicity island [3], a large segment of 43 Kb DNA flanked by direct repeats, that encodes a type III secretion system and the adhesin intimin. Intimin is necessary for maximal colonization by O157:H7 in mouse, pig, and calf models [4–7].

Cattle and other ruminants are the natural carriers of STEC O157:H7, and contamination of the organism in meat most often occurs during beef processing [8–10]. Undercooked ground beef was responsible for the majority of STEC outbreaks initially [11, 12]; however, contaminated fresh produce and non-pasteurized beverages also spread STEC [13–17]. After ingestion of even low doses of *E. coli* O157:H7, the most common disease manifestation is bloody diarrhea or hemorrhagic colitis (HC) [18]. A serious sequela of *E. coli* O157:H7 infection, the hemolytic uremic syndrome (HUS), is defined by thrombocytopenia, hemolytic anemia, and kidney failure and occurs in 10–20 % of individuals [18]. It is not known why some individuals spontaneously clear infection while others progress to the HUS. Infectious dose and age of the patient have been implicated [19–21]; however, these factors alone do not account for the total observed disease variance [18, 22].

Host genetic factors are likely important determinants for O157:H7-related disease outcome. Because traditional laboratory animal models are inbred to reduce genetic heterogeneity and to minimize variably between experiments [23], a different animal model was required to investigate phenotypic variations and to reflect the complex genetic structure of the human population. The advanced recombinant inbred murine (ARI) BXD panel [24], was created by intercrossing two mouse strains, C57BL/6 J (B6) and DBA/2 J (D2). The progeny BXD strains were inbred to homozygositiy and genotyped to determine the genotypic contest of each strain and to identify the location of crossover events. The BXD panel has approximately 580,000 single nucleotide polymorphisms (SNPs) and microsatellite markers [25, 26]. In addition, the genomes of the parental parents B6 [27] and D2 [28, 29] were sequenced and found to differ at approximately 4.8 million SNPs. Phenotypic differences among the BXD strains are primarily related to the SNPs and 500,000 insertion-deletions [26].

Therefore, use of the BXD panel allows for a systems genetic, genome-wide analysis to facilitate identification of a quantitative trait locus (QTL) responsible for phenotypes such as variation of severity of diabetes [30], forebrain weight [31], bone density [32], or addiction response to alcohol [33, 34]. Of particular relevance to this study, the BXD panel was used to explore genetic traits that underlie the response to infectious diseases such as influenza [35], streptococcal sepsis [36, 37], and Ebola [38] infections. A comparison of the human and murine genome reveals a high degree of similarity [39]. Therefore, it is theoretically possible to translate QTL findings from BXD mice to humans (reviewed in [40, 41]). Thus, we theorized that host genetic loci that impact colonization by O157:H7 in the BXD panel may reflect human traits responsible for STEC disease.

In this study, we observed a statistically significant difference in colonization levels in the murine parental strains (B6 and D2) after infection with TUV86-2. That difference indicates the presence of a potential QTL involved in O157:H7 colonization that may be masked when Stx2a is expressed by the infecting *E. coli* O157:H7 strain. Analysis of colonization data from 24 BXD strains infected with TUV86-2 identified a highly significant QTL on proximal chromosome (chr) 9 between 12.5 and 26.7 Mb that strongly predicts variation in colonization levels one day post-infection, accounting for 15–20 % of variance. This QTL harbors several genes known to regulate immune responses to bacterial infections. We evaluated candidate genes within this QTL using multiple parameters that included linkage, gene ontology, variation in gene expression, co-citation networks, and biological relevance. We identified five genes of interest that may be responsible for the observed differential colonization phenotype.

## Methods

### Ethics statement

All animal studies were approved by the Institutional Animal Care and Use Committee of the Uniformed Services University of the Health Sciences and were conducted in strict accordance with the recommendations of the Guide for the Care and Use of Laboratory Animals [42]. Animals were housed in filter top cages with access to food and water *ad libitum* unless otherwise noted, in an environmentally controlled room approved by the American Association for Accreditation of Laboratory Animal Care (AAALAC).

### Mice

Female mice, approximately 5–6 weeks old were used for all experiments. BXD parental strains (B6 and D2) were purchased from The Jackson Laboratory (JAX) (Bar Harbor, Maine). We obtained some BXD strains through collaboration with investigators at the University of Cincinnati (UC) who had acquired BXD breeding pairs from the University of Tennessee Health Science Center (UTHSC) (Memphis, Tennessee) [24]. Ten BXD strains (BXD 32, 44, 49, 51, 55, 65a, 75, 86, 96, and 98) were only analyzed from UC. Additional BXD strains were from UC or JAX. Similar colonization levels between mice from UC and JAX were confirmed with four BXD strains (BXD #: 73, 73b, 83, 87) (Additional file 1: Figure S1). Ten additional BXD strains (BXD #: 45, 48, 61, 62, 66, 69, 70, 84, 100, and 101) were tested from both UC and JAX (Additional file 1: Figure S1), while five BXD strains (BXD #: 60, 71, 73a, 99, 102) were only analyzed from the JAX colony. A minimum of two biological replicates were conducted for each BXD strain. We tested total of 31 strains, 29 BXD strains and ancestral parental strains B6 and D2, with 321 mice total (each BXD strain *n* = 6–20; B6 and D2 *n* = 48).

### *E. coli* O157:H7 strains and growth conditions

Colonization studies with the BXD parental strains (B6 and D2) were conducted with two STEC O157:H7 strains: 86–24, an Stx2a positive clinical isolate, and TUV86-2 an Stx2a-negative isogenic mutant [43–45]. BXD colonization studies were conducted only with TUV86-2. Both STEC strains are resistant to nalidixic acid (Nal) and were grown in Luria broth supplemented with 25 μg/mL Nal. To prepare the inoculum, an overnight culture (~18 h) was pelleted by centrifugation (5000 × g), the supernatant removed, and the pellet resuspended 1:100 in phosphate buffered saline (PBS) supplemented with 20 % sucrose. The inoculum was serially diluted and plated to determine the dose/mouse.

### Intact commensal flora (ICF) infection model

Colonization levels were determined in the ICF infection model as previously reported [46]. Briefly, food and water were removed from the mice for 20 or 2 h, respectively, prior to infection. Mice were fed a high inoculum, approximately 10^10^ colony forming units (CFU) in 100 μL by pipette tip. Each experiment included three mice per strain, with six to seven strains total. The parental B6 and D2 strains were included in all experiments as an internal control for colonization levels. Mice were weighed daily and colonization levels were reported as CFU per g feces on day one through day four post-infection.

To determine the CFU per g feces, mice were placed in individual cages with no bedding for 30–40 min. After this time, mice were returned to their original cage and fecal pellets were collected, weighed, and resuspended 1:10 w/v in PBS. The fecal slurry was further diluted 1:10 in PBS and plated on sorbitol MacConkey (SMAC) agar supplemented with Nal to select for the inoculating strain. The dilution that contained between 30 and 300 colonies was counted to determine CFU per g feces. The limit of detection for this model is 100 CFU per g.

### Data analysis and QTL mapping

We dedicated a considerable amount of time to data error checking and filtration after each experiment. Data from individual mice were flagged in the database and excluded from the final analysis if there were factors that affected colonization levels other than infection. For the final analysis we used data from 24 BXD strains and the parental mice. We performed general linear model (GLM) analysis of covariates using ordinary least-squares analysis of variance (OLS ANOVA) to determine the relative effect and interactions of covariates on the genetic factor, represented as mouse strain. GLM analysis revealed that there were no differences associated with mouse age, source, or the seasonality of the experiment (*P* > 0.15), Additional file 2: Table S1. Mouse strain was the most significant predictor of colonization (*P* <0.0001), followed by inoculum (*P* = 0.02). Although the differences in inoculum were statistically significant, we do not believe them to be biologically significantly, as they were within the same log for all experiments and did not result in any difference in colonization levels of the parental strains. We used the open-access web-based interval analysis on the GeneNetwork (GN) platform for complex trait analysis to identify QTLs. The primary data has been entered under trait IDs 17467, 16607, 16608, 18071, and 18072. The genome-wide interval mapping module allowed us to analyze phenotypes in the context of mouse genotypic differences and estimate the significance at each location using 5000 permutation tests [25]. We did ten sets of analyses of the log CFU means, log CFU medians or corrected coefficient of log CFU/g feces for the following variables: 1) colonization one day post-infection; 2) colonization two days post-infection; 3) colonization 3 days post-infection; 4) colonization 4 days post-infection; 5) difference in colonization between day four and one post-infection; 6) difference in colonization between day four and two after infection; 7) difference in colonization between day four and three after infection; 8) difference in colonization between day three and one after infection; 9) difference in colonization between day three and two after infection; and, 10) difference in colonization between day two and one after infection, for 30 traits analyzed. In addition, we determined the overall variation in median colonization across the BXD panel over time from the linear and polynomial slopes of colonization change per strain. Therefore 32 traits overall were mapped for QTLs.

## Bioinformatic analyses of mapped QTLs

### Haplotype analyses of significant QTL on Chr 9

We analyzed haplotypes of the BXD strains used in this study at the significant mapped QTL on Chr 9 between 12.6 and 25.6 Mb. We downloaded the BXD genotype data set as a Microsoft excel file from http://www.genenetwork.org/genotypes/BXD.geno and selected for the Chr 9 mapped QTL region and examined the genotypes of the BXD strains from that database. The different BXD strains were rank-ordered according to colonization levels on day one post-infection.

### Polymorphism analysis (SNP analysis)

We did SNP analysis on genes and transcripts in the mapped QTLs associated with STEC O157:H7 differential colonization. The analysis was done on the 24 BXD strains and the parental strains B6 and D2. SNP analysis data were retrieved on March 22, 2015 from the mouse phenome database (MPD) with the SNP wizard online tool at http://phenome.jax.org/SNP. The retrieved data originated from several databases: national center for biotechnology information (NCBI) Mouse Build 37 (known as mm9), NCBI SNP database (dbSNP) build 128 and the joint project of the European Molecular Biology Laboratory (EMBL), European Bioinformatics Institute (EBI) and the Wellcome Trust Sanger Institute, (Ensembl) build 48. SNPs among BXD strains were based on Wellcome-CTC Mouse Strain SNP Genotype Set [26].

### QTL heatmap

We did correlation analyses of all traits associated with the differential colonization parameters described in the QTL analysis section above (12 traits) with QTL heatmap. QTL heatmap is a bioinformatics tool offered by the GeneNetwork (GN) platform in which a hierarchical cluster tree of the mapped set of traits is computed and distances between pairs of traits is calculated with the formula1-r, where r is the Pearson product–moment correlation.

### Candidate gene co-citation analysis

We did co-citation analyses using open-source Chilibot platform (www.chilibot.net), a literature search engine that identifies all relevant relationships among search terms [47]. We cross-referenced the 36 candidate polymorphic genes using key words associated with STEC O157:H7 colonization to identify genes most likely responsible for the QTL on Chr 9. The initial key words were: STEC; colonization; normal flora; colonization resistance; epithelial cell; tight junction; cell polarity; adhesion; mucus; mucin; nucleolin. The key words for the final interaction diagram, chosen because of the high degree of interconnectivity to the top genes and for clarity of the figure were: STEC; colon; mucus; colonization.

### Statistical analysis

All primary calculations and sorting operations were done with features and functions of Microsoft Excel. Statistical analyses were executed with Data Desk (version 6.3) software (Data Description, Inc., Ithaca, NY; www.datadesk.com) and included correlation, regression, and general linear model (GLM) analyses by ordinary least squares analysis of variance (OLS ANOVA). Mouse age, weight, inoculum, and log CFU/g feces for each day post-infection were analyzed to determine the possible effect of those variables on the final results.

### Data deposition

Our data sets were stored as part of the BXD published phenotypes on Gene Network Platform and can be found at (www.genenetwork.org) under BXD published phenotypes record trait IDs 17467, 16607, 16608, 18071, and 18072.

## Results

### Infection with TUV86-2, but not 86–24, results in significant colonization differences between the parental murine strains

**Fig. 1:**
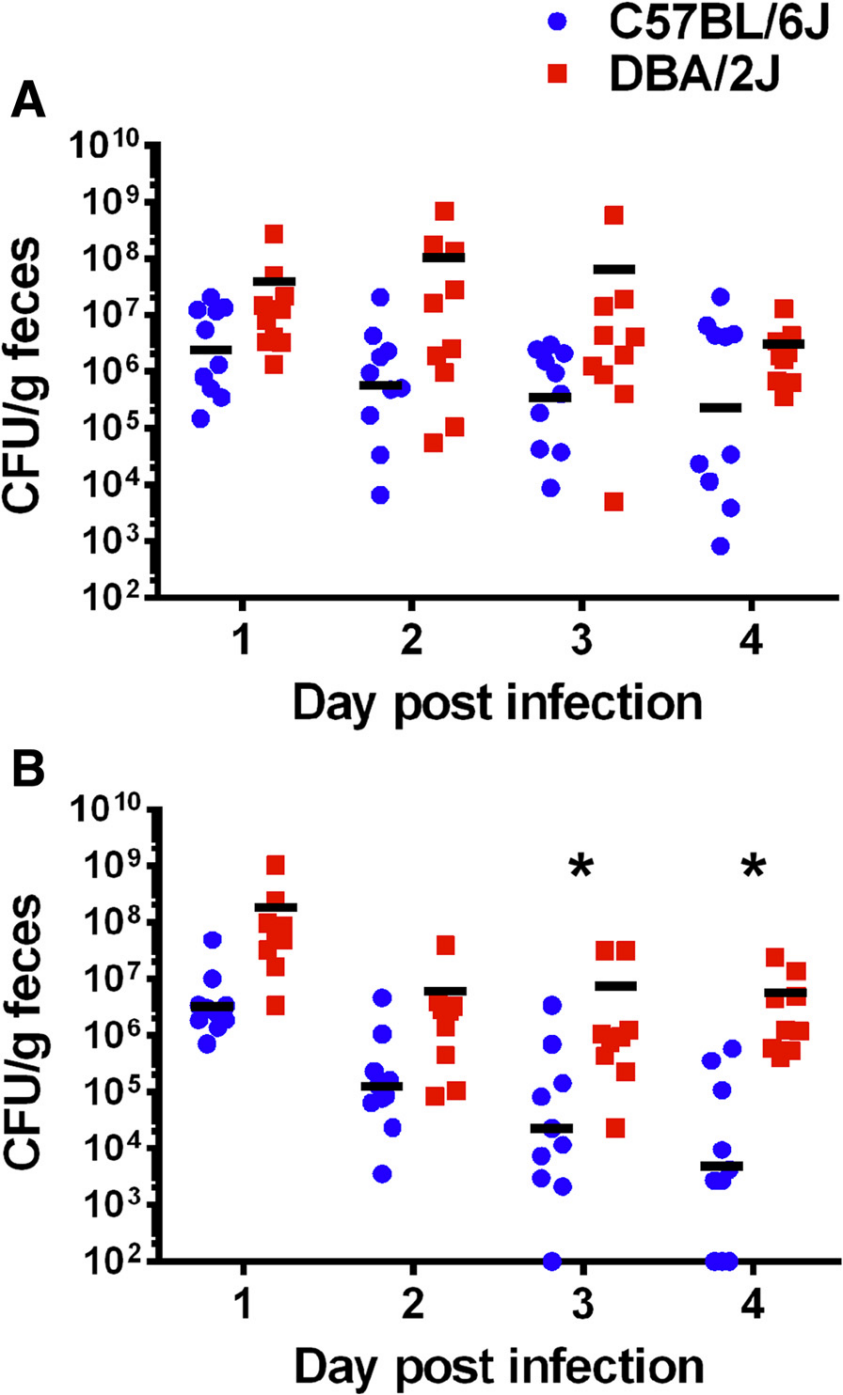
Colonization levels in BXD parental strains after infection with STEC O157:H7 strains. B6 and D2 strains were infected with isogenic O157:H7 strains 86–24 (Stx2a+) (**a**) or TUV86-2 (Stx-) (**b**). Individual mouse colonization levels are depicted as CFU/g feces over the course of the experiment and the black bars represent the geometric mean of the group. (*) The difference in colonization levels between B6 and D2 mice was significant after infection with TUV86-2 on days 3 and 4 as D2 mice maintained colonization while B6 showed reduced colonization or even cleared the infection (*P* ≤ 0.003). *n* = 10. Limit of detection was 10^2^ CFU/g

We used the ICF infection model to determine the colonization levels of two isogenic STEC O157:H7 strains in the BXD parental mice. After infection with 86–24, an Stx2a-positive strain, both B6 and D2 mice had an average colonization level of 10^6^–10^7^ CFU/g feces on day one post-infection (Fig. 1a). The colonization levels did not vary significantly between B6 and D2 over the course of the experiment, and both murine strains maintained colonization through day 4. We next determined colonization levels from B6 and D2 mice infected with TUV86-2, an Stx2a-negative isogenic mutant. The initial TUV86-2 colonization levels were similar to those after infection with 86–24: the geometric mean colonization levels of the parental B6 and D2 murine strains was 10^6^ or 10^8^ CFU/g feces, respectively (Fig. 1b). However, a significant difference in colonization levels developed by day three post-infection, such that D2 mice maintained colonization while B6 mice began to resolve the infection (Fig. 1b). The significant difference in colonization was maintained on day 4.

### Colonization differences between parental and BXD strains infected with TUV86-2

**Fig. 2:**
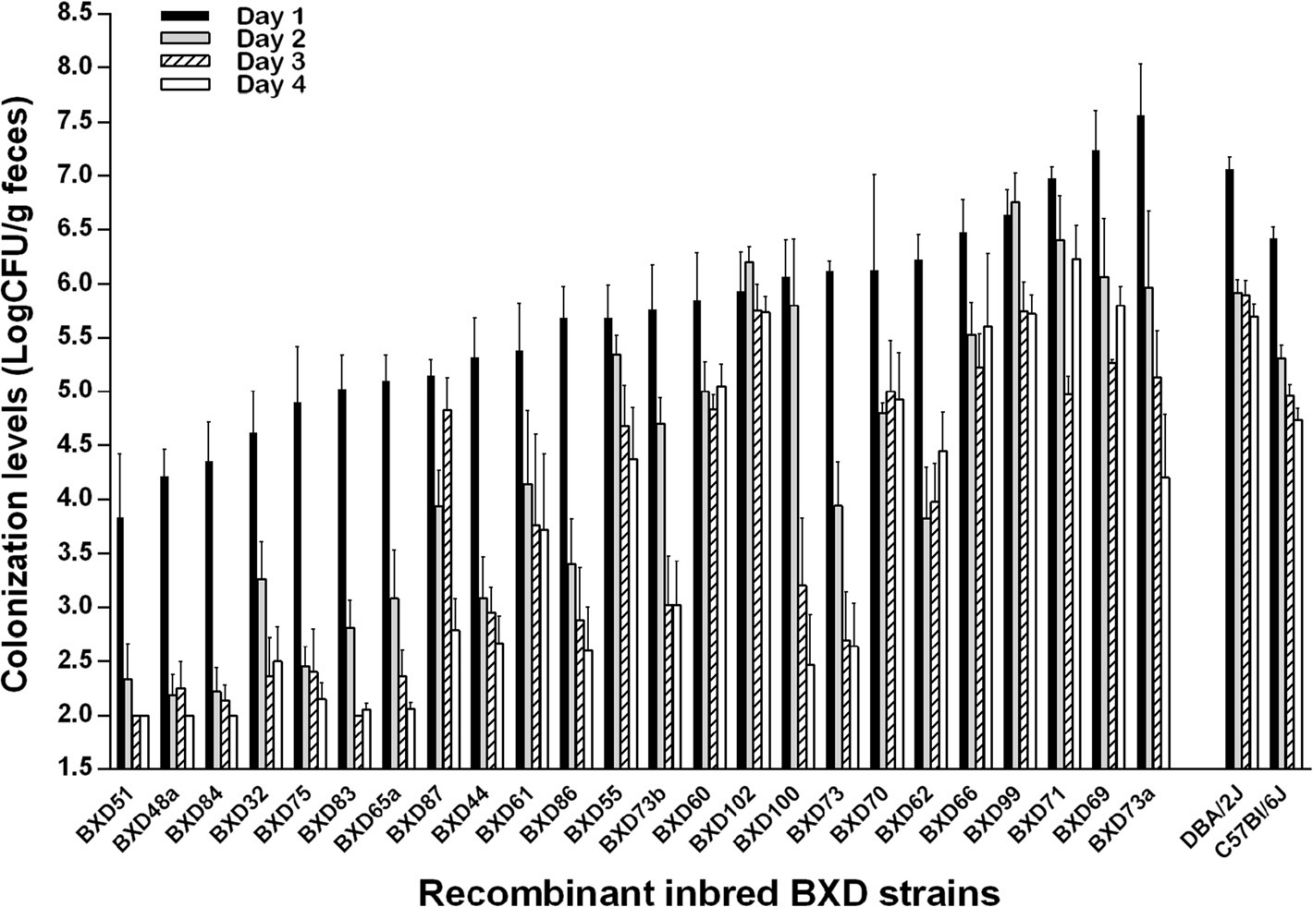
BXD colonization levels after infection with TUV86-2. The TUV86-2 colonization levels for the BXD and parental murine strains over the course of the infection. Individual murine strains (sorted based on day one colonization from lowest to highest) are listed along the x-axis and daily colonization levels are depicted as the log CFU/g feces. Parental *n* = 31; BXD *n* = 3–9 per strain; 182 mice total. Limit of detection was 10^2^ CFU/g

Since there was a significant difference in the colonization levels of the parental mice after infection with TUV86-2, we decided to infect the BXD mice only with TUV86-2. All of the BXD strains tested became colonized with TUV86-2 after oral inoculation with the organism. Although the mean colonization levels of the parental murine strains one day post-infection were 1.38 × 10^6^ or 1.15 × 10^7^ CFU/g feces, respectively, for B6 and D2 mice, the mean colonization levels from the different BXD strains one day post-infection ranged from 10^4^ to 10^7^ CFU/g feces (Fig. 2). Additionally, individual BXD strains exhibited different patterns of colonization over the course of the infection. A few strains maintained colonization (BXD 99 and 102), others steadily lost colonization (BXD 51, 75, 96, 97), and some others showed variable colonization over the experiment (BXD 60, 62, 71, 87, 100) (Fig. 2). These data demonstrate variable susceptibility to O157:H7 colonization within the BXD panel and suggest that colonization levels might be used to identify host genetic factors associated with the capacity of STEC to establish infection.

### QTL identified on proximal Chr 9 associated with TUV86-2 colonization in BXD mice

**Fig. 3:**
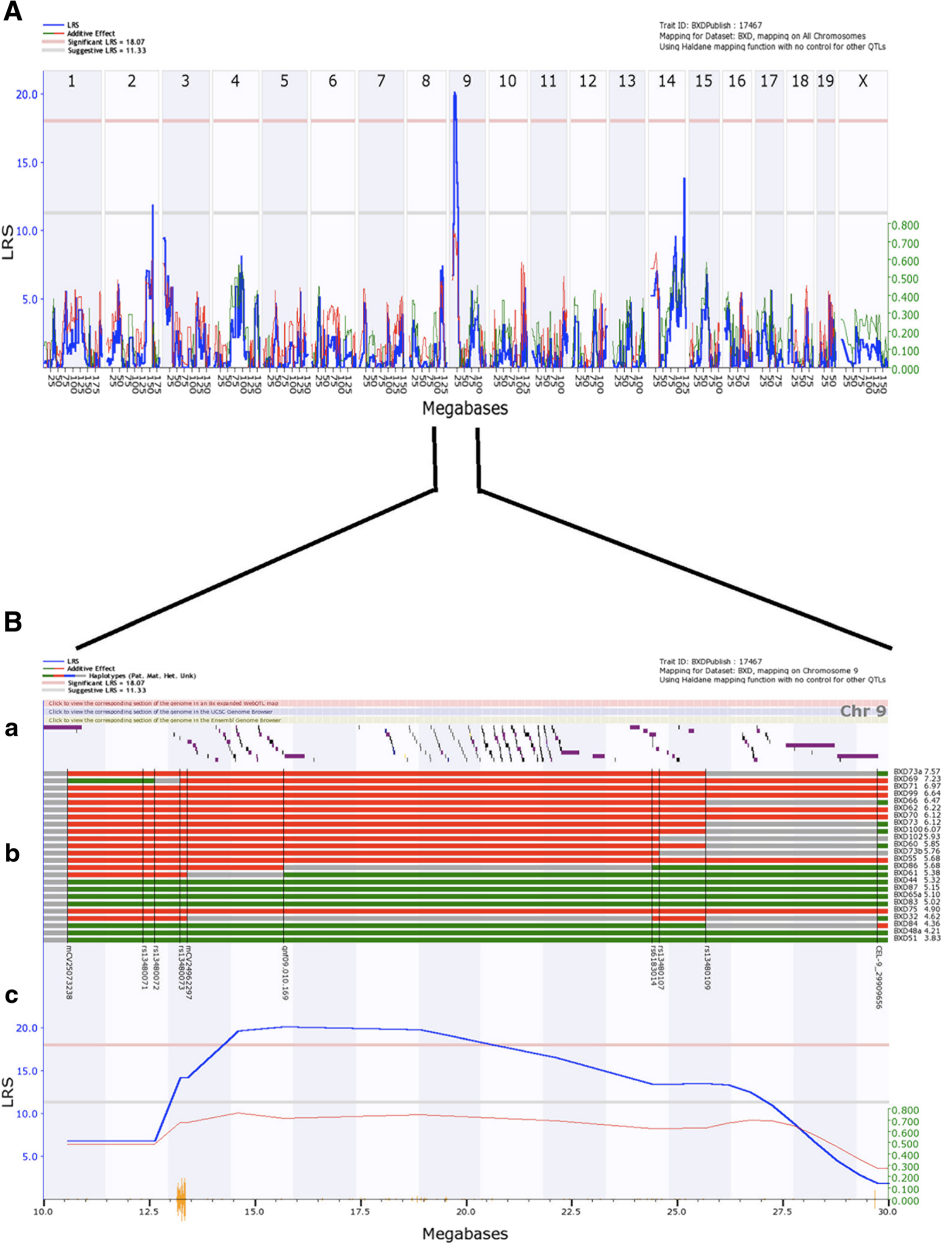
Genome wide scan for TUV86-2 susceptibility revealed a significant QTL on chromosome 9. **a**) A mouse chromosome interval map based on TUV86-2 colonization levels on day one. The x-axis depicts the physical map of the entire murine genome. The left y-axis illustrates the LRS (blue line) as an expression of strength of the association between colonization and genotypic markers. The grey line at *y* = 11.25 indicates the suggestive threshold, while the pink line at *y* = 17.88 shows the significant threshold. A significant QTL was identified on proximal Chr 9. **b**) Expanded physical map of Chr 9 for the region of the QTL. (**a**) Colored blocks represent the location of individual genes along the Chr. with links to corresponding section of the genome in the UCSC Genome Browser, the Ensembl Genome Browser, and expanded WebQTL map. (**b**) Haplotype map of BXD strains (listed on the left side with the log geometric mean colonization on day one listed next to the strain name), where green denotes D2 (paternal), red designates B6 (maternal), blue shows heterozygous and grey indicates an unknown genotype. Genetic markers associated with the mapped QTLs are shown: proximal mCV25073238, rs13480072, rs13480071, rs13480073, mCV24962297, gnf09.010.169 (associated with highest LRS), rs6183014, rs13480107, rs13480109 and CEL-9_29909656. (**c**) Expanded view of the QTL (blue line) overlaid on the SNP seismograph track, where each orange hash mark indicates a unique SNP. The right y-axis represents the additive allele effect and the red line signifies that the B6 allele is associated with increased colonization levels

We performed genome-wide scans with bioinformatics tools provided by GeneNetwork (www.genenetwork.org) to assess the observed colonization levels against the known genotypes of the BXD strains. We analyzed TUV86-2 colonization levels in the BXD strains by the 32 parameters listed in the methods. We identified a significant QTL on proximal Chr 9 when we mapped the log of the colonization means from day 1 (Fig. 3a), with a likelihood ratio statistic (LRS) of 20.19 [limit of detection (LOD) = 4.4 and *P* < 0.05] and a total interval width of 14 Mb (12.5–26.7 Mb) (Fig. 3b). We next did linkage analysis of the QTL on proximal Chr 9 and found that the QTL was linked with three genetic markers, gnf09.010.169, rs13480073, and mCV24962297 (13.23–15.69 Mb), with a peak LRS at 15.69 Mb associated with genetic marker gnf09.010.169. When we mapped colonization levels on day 2 or 4 post-infection, we found a suggestive QTL that overlapped the Chr 9 QTL for day one colonization at interval 13–26 Mb (Table 1).

**Table 1:** Summary of TUV86-2 colonization QTLs in ARI BXD mice^a^

Name of mapped trait (Phenotype)	Chr (mm9^b^)	Peak LRS	Genetic marker(s) associated with peak locus	Location of genetic markers (Mb)
Colonization one day after infection	9	20.19^c^	gnf09.010.169, rs13480073, and mCV24962297	13.23–15.69
	14	13.84	rs13482392, gnf14.114.290, rs13482396	118.97–119.71
Colonization two days after infection	9	16.12	gnf09.010.169	13.23–25.69
	14	12.09	rs13482396, gnf14.085.610 and rs3707842	91.32–120.03
Colonization four days after infection	9	12.29	gnf09.010.169, rs13480073, mCV24962297	13.23–25.69
Difference in colonization between day two and one after infection	13	12.05	rs6209128 and rs3023086	52.86–53.52
Difference in colonization between day three and one after infection	7	13.36	rs6206014	47.86
	18	11.98	rs3718618 and rs3669949	69.37–69.9
	19	12.99	rs13483513, gnf19.005.316, rs4232041 and rs4232042	3.41–10.15
Difference in colonization between day four and one after infection	5	12.86	rs13478413 and rs3688859	98.23–100.05
	17	11.6	rs13483110	76.56
Difference in colonization between day three and two after infection	4	15.52	rs3719891–rs6358921	140.88–150.45
	10	12.04	rs13459120, rs13480580	35.75–36.17
	15	12.02	rs4230714–rs3701428	44.2–55.00
Difference in colonization between day four and two after infection	1	11.66	rs13475818 and UT_1_38.719268	38.07
	15	11.28	rs3717268 and rs13482709	90.7–91.5
	X	16.64	rs13483746, rs13483748 and rs13483736	44.68–48.08
Difference in colonization between day four and three after infection	12	11.17	rs13481566–rs13481579	85.87–89.93
Overall strain specific variation in pattern of colonization across BXD strains (Linear slopes of medians)	17	12.47	rs13483110	76.5
Overall strain specific variation in pattern of colonization across BXD strains (Polynomial slopes of medians)	X	12.17	rs13483770, gnfX.044.260, rs13483785, rs13483786	56.48–61.86

^a^Trait linkage analysis done with 5000 permutation tests

^b^mm9: NCBI Mouse Build 37

^c^Significant quantitative trait locus

We also identified suggestive QTLs that overlapped on Chr 14 for colonization levels on days one or two post-infection with a peak LRS of 13.84 and 12.09, respectively (Table 1). We further identified multiple suggestive QTLs for the following traits: difference in colonization between two independent days post-infection [such as colonization day two minus colonization day 1 (QTL on Chr 13)], and the linear (Chr 17) and polynomial slopes of colonization change (Chr X) (Table 1).

**Fig. 4:**
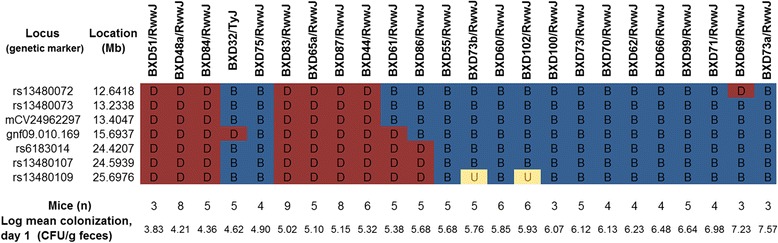
Haplotype of BXD strains within the region of the QTL on Chr 9. The BXD strains are arranged in order from the lowest to highest colonization levels one day post-infection. The overall strain distribution pattern is that the D2 haplotype (D, red) is associated with low colonization, while the B6 haplotype (B, blue) is associated with high colonization. U indicates an unknown genotype

We identified the haplotypes of the BXD strains at the significant QTL on Chr 9 between 12.6 and 25.6 Mb and rank-ordered BXD strains according to colonization levels from low to high (Fig. 4). Strain distribution patterns (SDP) of the BXD strains revealed that high colonization levels on day one post-infection were associated with the B allele (blue) inherited from the parent B6. Low colonization levels in the BXD panel were associated with D alleles (red) inherited from the D2 parent. Taken together the SDP of the haplotypes suggests that overall the B allele exhibited dominance for high colonization. In addition, we performed QTL heatmap analysis that entailed correlation analyses for 12 traits associated with differential colonization (Additional file 3: Figure S2). The phylogenetic tree at the top of the QTL heatmap indicates how closely related the independent traits are to each other. We observed that the significant mapped QTL on Chr 9 was associated with B allele dominance (dark blue) in accordance with haplotype analyses. Other mapped QTLs on Chrs 1 and 5 had similar B allele dominance. In contrast, QTLs on Chrs 14, 15 18, and 19 had D allele dominance (Additional file 3: Figure S2).

### Candidate genes analyses

**Fig. 5:**
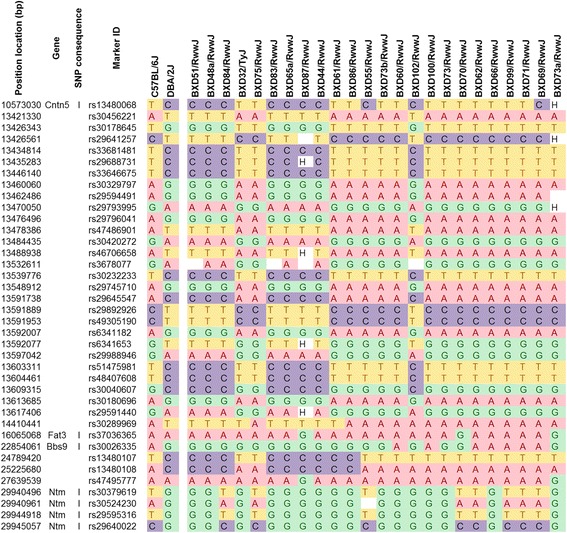
Selected SNPs from the Chr 9 QTL. The SNPs of the parental and BXD strains are identified at 38 nonsynonymous loci of interest. The parental strains are shown first and the BXD strains are listed in order from lowest to highest colonization levels one day post-infection. Low colonization was associated with the D2 haplotype and high colonization was associated with the B6 haplotype. SNP consequence “I”: SNP occurs within an intron. Haplotype “H”: heterozygous; blank white box: unknown

**Fig. 6:**
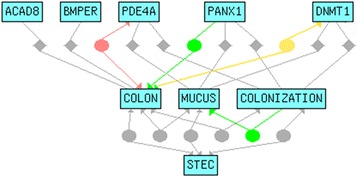
Gene interaction analysis of the five genes predicted to be responsible for the Chr 9 QTL. The final 4 key words (STEC; colonization; colon; mucus) and the interactions between and among the five genes predicted to be important for the QTL: Acad8; Bmper; Pde4a; Panx1; Dnmt1. Circles indicate an interactive relationship while diamonds indicate that a co-occurrence was found. A green line indicates a stimulative relationship; a red line signifies an inhibitory relationship; a yellow line shows both stimulative and inhibitory relationships; and a grey line denotes a neutral relationship. The Chilibot search was conducted on April 9, 2015. There were nine search terms and 55 searches were conducted. A total of 2364 PubMed records were searched with 239 (10.1 %) records processed and 20 links were found

We did gene enrichment analyses of the significant QTL mapped on Chr 9 with multiple parameters that included linkage, gene ontology, variation in gene expression, polymorphism, co-citation networks, and biological relevance. Polymorphism (SNP) analysis identified 36 candidate genes that might modulate differential colonization associated with the identified QTL on proximal Chr 9. SNPs were identified by the Mouse Phenome Database (http://phenome.jax.org/). We focused on nonsynonymous SNPs, even those located within exons since those SNPs may influence translation. We found 38 SNPs of interest (Fig. 5) and with the ToppGene suite (https://toppgene.cchmc.org/) we identified 36 candidate genes (Table 2). Finally, we did co-citation networks and biological function analyses for candidate genes and key words (listed in methods). Through those analyses, we identified five genes that are most likely to modulate differential colonization. These are Pannexin 1(Panx1); BMP binding endothelial regulator (Bmper); DNA methyltransferase 1 (Dnmt1); phosphodiesterase 4A (Pde4a); and acyl-CoA dehydrogenase family, member 8 (Acad8). A visual representation of the relationship between the final key words (STEC; colonization, mucus, colon) and the five genes of interest is shown in Fig. 6.

**Table 2:** Candidate genes on Chr 9

Gene symbol	Name or description	Chr location (GRCm38)	Size	Identifiers
Cntn5	Contactin 5	9:9660891–10904775 –	1243884	MGI:3042287
Maml2	Mastermind like 2 (Drosophila)	9:13619990–13709388 +	89398	MGI:2389460
Sesn3	sestrin 3	9:14276301–14326138 +	49837	MGI:1922997
Amotl1	Angiomotin-like 1	9:14541966–14615483 –	73517	MGI:1922973
Mre11a	Meiotic recombination 11 homolog A (S. cerevisiae)	9:14784654–14837123 +	52469	MGI:1100512
Panx1	Pannexin 1	9:15005161–15045478 –	40317	MGI:1860055
4931406C07Rik	RIKEN cDNA 4931406C07 gene	9:15283337–15306448 –	23111	MGI:1918234
Taf1d	TATA box binding protein (Tbp)-associated factor, RNA polymerase I, D	9:15306214–15312104 +	5890	MGI:1922566
Ccdc67	coiled-coil domain containing 67	9:15559864–15627914 –	68050	MGI:2443026
4930540M03Rik	RIKEN cDNA 4930540 M03 gene	9:15619857–15641220 +	21363	MGI:1925275
Fat3	FAT tumor suppressor homolog 3 (Drosophila)	9:15910205–16378231 –	468026	MGI:2444314
Naalad2	N-acetylated alpha-linked acidic dipeptidase 2	9:18321951–18402995 –	81044	MGI:1919810
Olfr39	Olfactory receptor 39	9:20282351–20286648 +	4297	MGI:1313142
Olfm2	Olfactomedin 2	9:20667986–20728219 –	60233	MGI:3045350
Col5a3	Collagen, type V, alpha 3	9:20770050–20815067 –	45017	MGI:1858212
Dnmt1	DNA methyltransferase (cytosine-5) 1	9:20907206–20959888 –	52682	MGI:94912
Pde4a	Phosphodiesterase 4A, cAMP specific	9:21165714–21213248 +	47534	MGI:99558
Ilf3	Interleukin enhancer binding factor 3	9:21368019–21405361 +	37342	MGI:1339973
Carm1	Coactivator-associated arginine methyltransferase 1	9:21546894–21589487 +	42593	MGI:1913208
Dock6	Dedicator of cytokinesis 6	9:21800184–21852635 –	52451	MGI:1914789
Gm6484	Predicted gene 6484	9:21835510–21837346 +	1836	MGI:3643534
Zfp599	Zinc finger protein 599	9:22247430–22259895 –	12465	MGI:2679006
Zfp810	Zinc finger protein 810	9:22276748–22307638 –	30890	MGI:2384563
Bbs9	Bardet-Biedl syndrome 9 (human)	9:22475715–22888280 +	412565	MGI:2442833
Bmper	BMP-binding endothelial regulator	9:23223076–23485202 +	262126	MGI:1920480
Npsr1	Neuropeptide S receptor 1	9:24097996–24316398 +	218402	MGI:2441738
Dpy19l1	dpy-19-like 1 (C. elegans)	9:24411776–24503140 –	91364	MGI:1915685
Dpy19l2	dpy-19-like 2 (C. elegans)	9:24557048–24696293 –	139245	MGI:2444662
Tbx20	T-box 20	9:24720812–24774303 –	53491	MGI:1888496
Sept7	Septin 7	9:25252439–25308571 +	56132	MGI:1335094
Eepd1	Endonuclease/exonuclease/phosphatase family domain containing 1	9:25481547–25604110 +	122563	MGI:1914734
Gm1110	Predicted gene 1110	9:26879567–26923081 –	43514	MGI:2685956
Acad8	Acyl-Coenzyme A dehydrogenase family, member 8	9:26974135–26999566 –	25431	MGI:1914198
Ncapd3	Non-SMC condensin II complex, subunit D3	9:27030175–27095311 +	65136	MGI:2142989
Opcml	Opioid binding protein/cell adhesion molecule-like	9:27790775–28925410 +	1134635	MGI:97397
Ntm	Neurotrimin	9:28994750–29963141 –	968391	MGI:2446259

## Discussion

The major finding from this study was the identification of a significant QTL on proximal Chr 9 associated with TUV86-2 colonization levels in BXD mice one day post-infection. The identification of this QTL supported our hypothesis that host genetics affect STEC O157:H7 colonization levels in mice. Since establishment of infection is critical for comparison of colonization levels across multiple experiments, we included the BXD parental strains in every experiment as an internal control. Because the B6 and D2 day one colonization levels were consistently within the expected range (10^6^–10^8^), we are confident that the variation in BXD colonization levels is due to genotypic differences among the strains. The variation in colonization levels across BXD strains is consistent with the mosaic genotypes of the panel. We predict that such genetic predispositions to low or high initial colonization levels could influence the severity of disease from an STEC infection. The mosaic-like genetic complexity of the ARI BXD panel provided the diversity required to map the QTL and would allow us to predict if an individual animal would be susceptible or relatively resistant to O157:H7 colonization.

We initially determined the colonization profiles of the parental murine strains after infection with both 86–24, an Stx2a-positive strain, and TUV86-2, an Stx2a-negative isogenic strain. However, since there was no difference in colonization levels of the parental strains after 86–24 infection, we decided to only infect the BXD strains with TUV86-2 to have the greatest chance of identifying a QTL associated with colonization. The reason that we did not observe a difference in colonization levels after infection by 86–24 is most likely due to the fact that Stx2a enhances STEC colonization levels in a traditional mouse model [45]. The decision to proceed with the toxin-negative strain enabled us to identify host genetic markers that may be associated with O157:H7 colonization. Future studies could determine whether the toxin positive strain would overcome the low colonization phenotype associated with the “D” allele at the Chr 9 QTL.

Suggestive QTLs linked to colonization levels on days two and four post-infection were also mapped to Chr 9 in the same region as the significant day one colonization QTL. In addition, an LRS that approached a suggestive QTL in the same Chr 9 region was mapped for colonization levels on day three post infection (data not shown), and the QTL heatmap for day three colonization also indicates B haplotype (blue color) dominance (Additional file 1: Figure S1). It is possible that the addition of more strains to the panel would strengthen the suggestive QTLs for colonization on days 3 and 4 post infection, time points for which days the parental mice also showed a difference in colonization. We believe that the overlap of multiple QTLs in one location bolsters the likelihood that this region of Chr 9 is tightly linked to colonization capacity. However, it is possible that different host factors are responsible for variations in colonization on day one compared to colonization persistence as measured on days 2 and 4. The additional suggestive QTLs identified in Table 1 linked to multiple traits illustrate the complexity of host genetic factors that respond to the presence of a bacterial pathogen. The QTLs associated with the differences in colonization between the days after infection are likely related to colonization persistence. That multiple QTLs on different Chrs are implicated in the persistence phenotype suggests that genes located across QTLs may be connected through similar pathways. This is especially evident by the three suggestive QTLs linked to the difference in colonization between day 3 and day 1 on Chrs 7, 18, 19.

Haplotype analysis in the region of Chr 9 that contains the significant QTL revealed that the D allele is associated with low colonization levels of BXD strains, a finding that contrasts with the colonization data from the parental strains in which the D allele was associated with high, sustained colonization. When there is such a reversal of SDP the QTL is referred to as a “cryptic” QTL, and is likely a reflection of the fact that the BXD panel was derived from the F2 progeny of the initial D2 x B6 cross [48]. It may be that the D2 have other factors that allow high colonization levels that mask the cryptic QTL on Chr 9. Future studies to test that latter hypothesis would determine the colonization levels of additional BXD strains that are “D” at the Chr 9 QTL to try to map a host factor(s) that allows high colonization in DBA but not the progeny.

We focused our QTL analysis on the significant QTL on proximal Chr 9. The QTL is located in a chromosomal region with limited SNPs. We chose the 36 candidate genes based on the highest number of polymorphisms, because such polymorphisms are more likely to be responsible for the QTL. We used Chilibot to compare scientific literature that included one or more of the 36 genes and key words associated with STEC colonization in the colon. Fig. 6 represents the final interconnectivity plot of the five genes most likely to be linked to the QTL: Acad8; Bmper; Pdea; Panx1; Dnmt1. All five of the genes selected are expressed in the colon of humans and mice. Acad8 is a member of the acyl-Coenzyme A dehydrogenase family and necessary for butyrate oxidation [49]. Butyrate, a short chain fatty acid, is an important source of energy for colonic enterocytes [50, 51] and helps to maintain intestinal epithelial cell physiology [52]. Defects in butyrate oxidation are linked to mucosal inflammation and ulcerative colitis [53]. Additionally, butyrate increases STEC adherence to CaCo2 cells [54] and increases the concentration of the Stx receptor, Gb3, on intestinal epithelial cells [55]. Polymorphisms of Acad8 may drive the QTL on Chr 9 by affecting the health of colonic enterocytes, which in turn promote or inhibit colonization.

Bmper, Pde4a, Panx1, and Dnmt1 are four genes that modulate inflammation of colonic enterocytes. Bmper is a BMP-binding endothelial regulator [49]. It limits endothelial inflammation by inhibiting expression of intercellular adhesion molecule 1 (ICAM1) [56, 57] and by regulating leukocyte extravasation and adhesion [58]. Pde4a is a cAMP-specific phosphodiesterase 4A that participates in multiple signal transduction pathways, such as platelet aggregation and immune cell activation [49]. Use of Pde4a-specific inhibitors has anti-inflammatory effects, such as reduced neutrophil adhesion [59] and inhibition of cellular trafficking and microvascular leakage [60]. Additionally, Pde4 inhibitors have been proposed to prevent STEC-mediated brain damage [61]. Panx1 is part of the innexin family, and as such a structural component of gap junctions [49] Panx1 is responsible for the release of ATP to the extracellular space, which can initiate cellular migration and inflammation [62]. Additionally, ATP release can modulate mucus secretion [63] which may impact colonization. Dnmt1 is a DNA methyltransferase responsible for the establishment and regulation of tissue specific methylated cytosine residues [49]. Since Dnmt1 activity affects global methylation patterns, variation in expression can change epithelial cell morphology [64]. Finally, Dnmt1 levels are elevated in response to UPEC infection [65]. Variation of inflammation levels could affect initial colonization, while the effect on leukocyte transit could impact colonization persistence. Further studies are needed to confirm or refute the actual involvement of one or more of the identified genes. The long term goal of this project is to correlate the genes identified within the murine QTL(s) with human host factor(s) responsible for the variable disease states observed during STEC O157:H7 outbreaks.

## Conclusions

We identified a QTL associated with colonization 1 day post-infection by O157:H7 on murine Chr 9. The identification of this QTL suggests that host genetics affect STEC O157:H7 colonization levels in mice

## Additional files

Additional file 1: Figure S1. Colonization levels of 14 BXD strains tested from JAX and UC. The individual colonization levels of mice from the 14 BXD strains that were tested from both JAX and UC are depicted. Mice from JAX are shown as red squares and mice from UC are shown as black circles. The colonization levels from both sources overlap, which supports the finding that there was no difference in colonization level depending on the source of the mice, Additional file 2: Table S1. (TIFF 320 kb) Additional file 2: Table S1. Analysis of variance for mean colonization day 1. (XLSX 40 kb) Additional file 3: Figure S2. Heat map of all mapped traits across the murine genome. The QTL heat map for all members of the cluster tree, from mouse Chr 1 to distal Chr X. The more intense colors mark chromosomal regions with comparatively high linkage statistics and the spectrum encodes the allelic effect. Each individually colored line in the vertical column indicates the genome-wide p value computed on the basis of 5000 permutations (significant p values are indicated by colors at the right end of the spectrum). (TIFF 258 kb)
