# Supplementary figures and images for: Mapping of genetic loci that modulate differential colonization by Escherichia coli O157:H7 TUV86-2 in advanced recombinant inbred BXD mice
Source: BMC Genomics. 2015 Nov 16;16:947. doi: 10.1186/s12864-015-2127-7 (PMC4647490; doi:10.1186/s12864-015-2127-7)

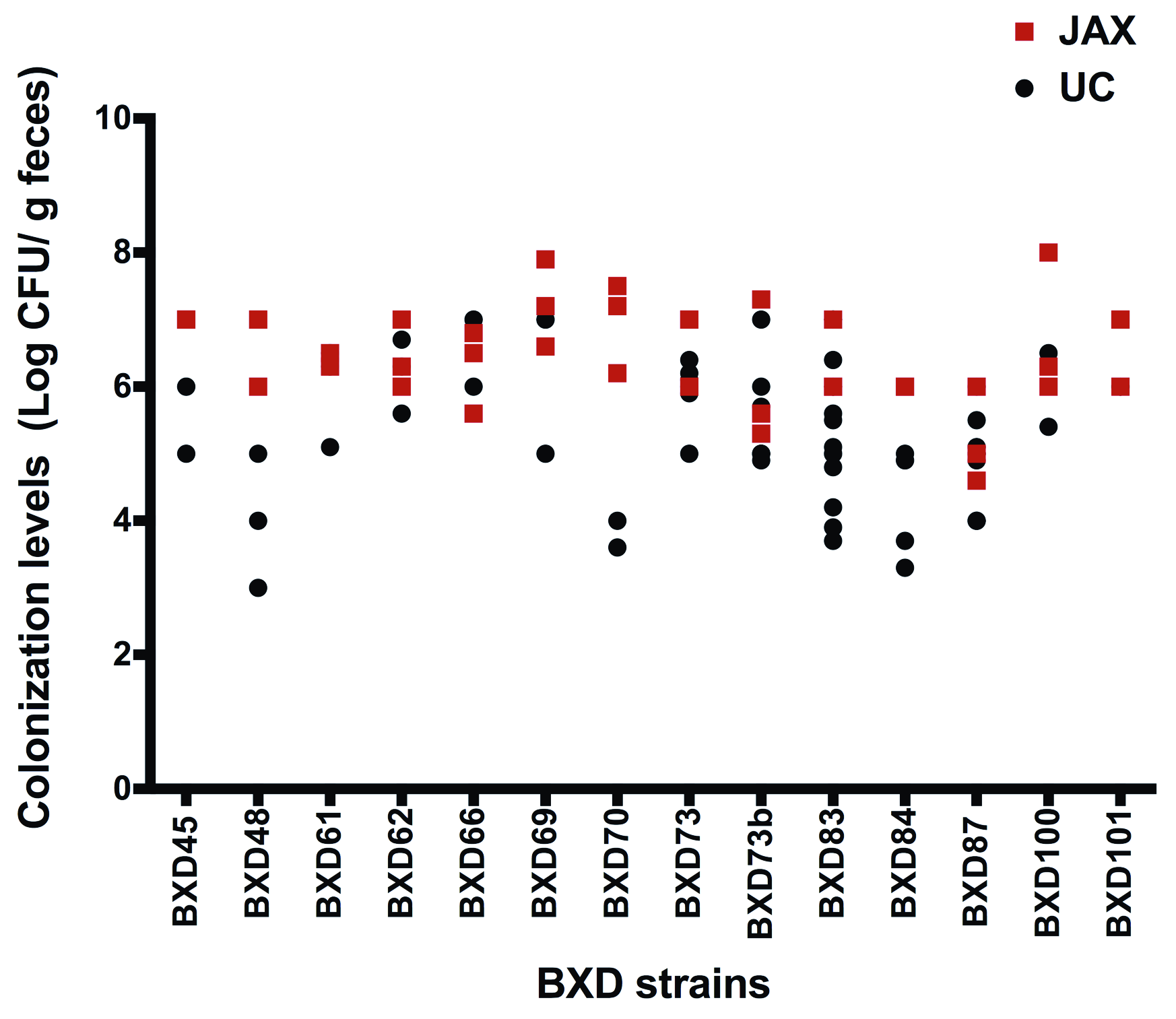

Supplement: Additional file 1: Figure S1. — Colonization levels of 14 BXD strains tested from JAX and UC. The individual colonization levels of mice from the 14 BXD strains that were tested from both JAX and UC are depicted. Mice from JAX are shown as red squares and mice from UC are shown as black circles. The colonization levels from both sources overlap, which supports the finding that there was no difference in colonization level depending on the source of the mice, Additional file 2: Table S1. (TIFF 320 kb) [file 12864_2015_2127_MOESM1_ESM.tiff]

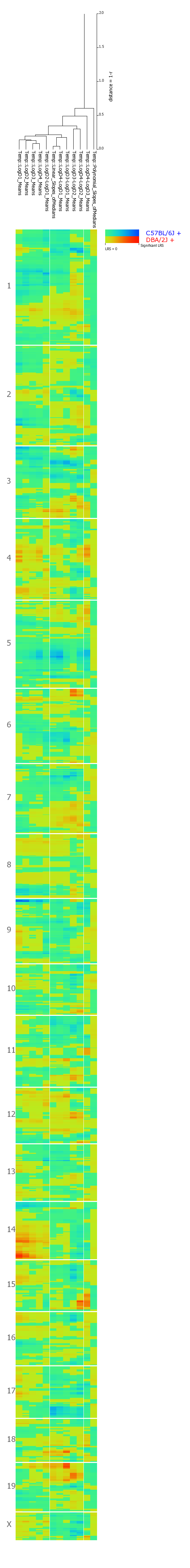

Supplement: Additional file 3: Figure S2. — Heat map of all mapped traits across the murine genome. The QTL heat map for all members of the cluster tree, from mouse Chr 1 to distal Chr X. The more intense colors mark chromosomal regions with comparatively high linkage statistics and the spectrum encodes the allelic effect. Each individually colored line in the vertical column indicates the genome-wide p value computed on the basis of 5000 permutations (significant p values are indicated by colors at the right end of the spectrum). (TIFF 258 kb) [file 12864_2015_2127_MOESM3_ESM.tif]
